# m^6^A facilitates YTHDF‐independent phase separation

**DOI:** 10.1111/jcmm.14847

**Published:** 2019-12-04

**Authors:** Si‐Yu Liu, Yi Feng, Jun-Jie Wu, Ming-Li Zou, Zi-Li Sun, Xia Li, Feng‐Lai Yuan

**Affiliations:** ^1^ Wuxi Clinical Medicine School of Integrated Chinese and Western Medicine Nanjing University of Chinese Medicine Wuxi China; ^2^ Department of Burns and Plastic Surgery The Affiliated Hospital of Jiangnan University Xingyuan Wuxi China

**Keywords:** m6A, methylation, phase separation, YTHDF protein

## Abstract

N6‐methyladenosine (m^6^A) is one of the most abundant messenger RNA (mRNA) modifications in eukaryotes and is involved in various key processes of RNA metabolism. In this issue of *Nature*, Ries et al (2019) described the fundamental features of m6A modification of mRNAs in regulating the composition of the phase‐separated transcriptome on the basis of number and distribution, and provide strong evidence that m6A plays a role in regulating phase separation in cells.

## 1 INTRODUCTION

Liquid‐liquid phase separation (LLPS) of biological macromolecules has rapidly become the focus of research in recent years. In 2009, Professor Tony Hyman of the Max Planck Institute in Germany discovered that P granules in nematodes were in fact a LLPS phenomenon of protein aggregation.^1^ Thereafter, this phase separation phenomenon was gradually verified as droplet formation caused by the collision and fusion of various cellular organelles; thus, some organelles are enclosed, whereas some are excluded from the droplets.^2^ This phenomenon is similar to the mixing of water and oil or can be imagined as the process of raindrop formation over an umbrella on rainy days. Scientists progressively discovered that many cellular membrane‐less organelles, such as nucleoli, Cajal bodies, stress granules, miRNA‐induced silencing complex and synaptic cytoskeletons, are phase transitions of specific proteins.^3^ Cells are separated from each other by phase separation to achieve a variety of biological functions. As soon as LLPS behaves abnormally, disease onset may occur; however, what triggered the aggregation of some molecules but not others in the same droplet has never been elucidated. It has been shown that biological macromolecules can form LLPS through multivalent interaction.^4^ The multivalent interaction of intracellular biological macromolecules is common and affected by some modifications, such as phosphorylation and methylation.^5^

**Figure 1:**
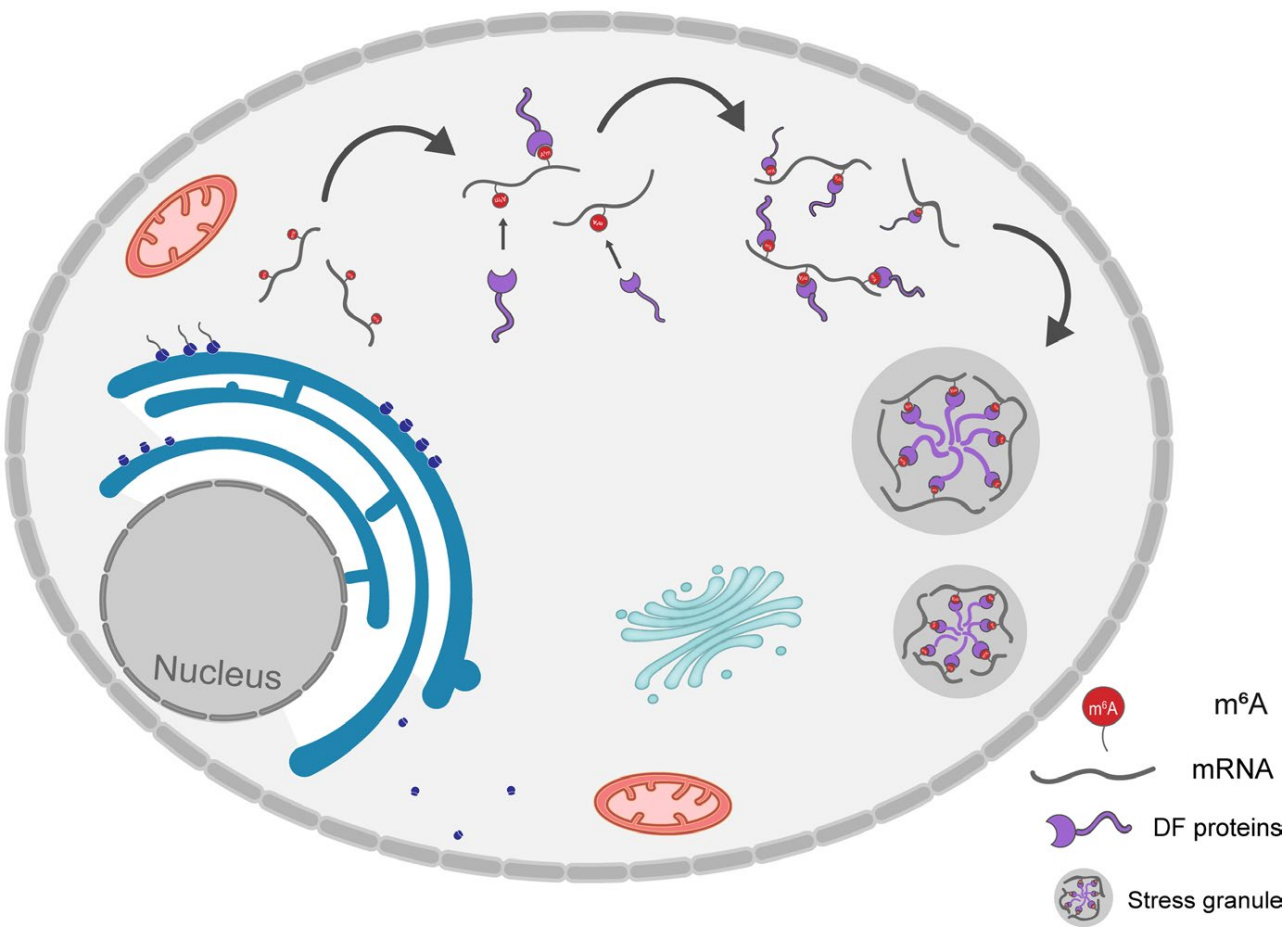
m^6^A acts as a ‘beacon’ to recruit DF protein into stress granules. The number and distribution of m^6^A sites in cytosolic mRNAs can regulate and influence the phase separation

Recently, Ries et al^6^ reported in *Nature* that the modification of m^6^A could enhance phase separation, and subsequently affected a series of cellular biological processes. The modification of m^6^A participates in the entire regulatory process of mRNA metabolism (specifically, modulating the stability of mRNA and determining cell differentiation), thus affecting various cellular biological processes and playing a crucial role in cell fate determination, lipid metabolism, and immunity. In mammalian cells, m6A modifications have been shown to be reversible, as it involves both methyltransferase and demethylase. Methyltransferase complex containing methyltransferase‐like 3 (METTL3), METTL14 and Wilms' tumour‐1–associated protein (WTAP) catalyses m6A methylation, whereas obesity‐associated protein (FTO) and AlkB homolog 5 (ALKBH5), the demethylases, catalyse demethylation of m6A. Moreover, m6A‐binding proteins with YTH domain, including cytoplasmic proteins YTHDF1, YTHDF2, YTHDF3, and nuclear protein YTHDC1, have been identified to be the ‘readers’ of m6A and modulate mRNA stability and translation to mediate downstream effects^7^ (Figure 1).

The authors selected the classic m^6^A‐binding proteins (YTHDF1 [DF1], YTHDF2 [DF2]. and YTHDF3 [DF3]) as research objects in this paper, thus attempting to reveal the specific regulatory mechanisms of m^6^A to the stability and translation efficiency of mRNA. Primary protein sequence analysis indicated that the YTHDF proteins consist of a m^6^A‐binding YTH domain of approximately 15 kD and a low complexity domain of approximately 40 kD. Based on this structural arrangement, the authors speculated that the three proteins may undergo LLPS. DF2 protein was the most abundant protein of DF paralogue purified from cells. In a low‐temperature environment of 4°C, the protein solution of DF2 was transparent. When the temperature elevated to 37°C, DF2 protein solution became turbid. When the temperature returned to 4°C, the solution turned again to clear, confirming that the DF2 phenomenon was LLPS. In the interim, it was also verified that DF2 protein at physiological concentrations could undergo phase separation. The authors additionally examined whether m^6^A modification could regulate the phase separation process of DF2 protein in vitro. The authors demonstrated that a 65‐nucleotide‐long RNA containing 10 modified m^6^A significantly enhanced the phase separation of DF proteins. Consequently, it is possible that m^6^A modifications regulate DF‐mediated clustering interactions involved in LLPS or granule formation.

To address whether m^6^A regulates the phase separation of DF2 protein, the authors found that in m^6^A methyltransferase 14–knockout mouse embryonic stem cells, the formation of endogenous stress granules took place normally after exposure to heat shock; however, the localization of DF2 proteins in stress granules was significantly reduced. The localization of DF2 to stress granules under cellular stress is dependent on its modification with m^6^A. Thus, the cellular localization of DF2 is modulated by the modification of m^6^A‐mRNA. When cells are exposed to heat shock, the translation efficiency of modified m^6^A‐mRNA in Mettl14‐knockout cells was markedly inhibited. Based on the above findings, the authors demonstrated that RNA modification by m^6^A, especially polymethylated mRNA, could bind to multiple DF2 proteins and significantly enhance the phase separation of DF protein induced by heat shock. These findings suggest that m6A controls the fate of cytosolic‐localized mRNAs by scaffolding DF proteins, which causes the formation of phase‐separated complexes that then partition into phase‐separated structures in cells. In addition, Xu et al^8^ recently demonstrated that an extensive amount of m^6^A‐modified mRNA was localized to stress granules when cells were exposed to oxidative stress. YTHDF was of vital importance for the formation of stress granules by modulating phase separation under oxidative stress. In the *BioRxiv* paper, the binding of YTHDF to m^6^A was inhibited, and the formation of stress granules was significantly reduced. Interestingly, the Nature article clearly reported that the knockout of METTL14 would not affect the stress granule formation after heat shock exposure. While in the BioRxiv paper, the binding of YTHDF to m^6^A was inhibited, and the formation of stress granules could be significantly reduced. That is to say, stress granule formation under oxidative and thermal stress may be triggered by different proteins, and the underlying precision regulatory mechanisms require further investigation. Almost simultaneously, in Cell Research, Li Pilong Research Group of Tsinghua University Life College published a paper entitled multivalent m^6^A motif promotion phase separation of YTHDF proteins, explaining the characteristics of m^6^A reader YTHDF1/2/3 and its potential function associated with the cellular response to stress from the perspective of LLPS.^9^ The three articles arrived at the same conclusion from different perspectives. Thus, m^6^A modification promotes the phase separation process of mRNA and its binding proteins.

This research offers experimental evidence for gene expression regulation by m^6^A modification through phase separation, and additionally provides a novel perspective and concept on the regulation of crucial gene expression in the process of cell fate determination and disease progression. Currently, the structures and mechanisms of organelles derived via phase separation are well‐understood, and the biological functions of phase separation will be the subject of further investigation. From the perspective of biochemistry, phase separation structures can selectively lead to molecule enrichment or repulsion, enhance the probability of molecular interactions and even alter the molecular conformation to promote or inhibit chemical reactions. Correlating these physical and chemical properties with the biological functions will be an important topic for future research. Employing m^6^A‐mediated phase separation as a target to modify cellular physiological processes, such as signal transduction, transcriptional regulation and stress response, will also provide a broad application spectrum for m^6^A‐mediated phase separation. Research on the association between phase separation and diseases is progressing promptly. Current research primarily focuses on neurodegenerative diseases.^10^ Protein point mutations, such as FUS, which can accelerate the transformation of regular liquid phase into solid phase, may be responsible for the formation of insoluble protein aggregates that are commonly observed in neurodegenerative diseases.^11^ Moreover, nucleus transport can reduce the concentration of nucleic acid‐binding protein in the cytoplasm, dissolve LLPS structures and prevent the formation of neurotoxicity caused by solid aggregation.^12^ It is expected that the research on phase separation–related diseases will be extended from neurodegenerative diseases to cancer and immunity. Investigation on the m^6^A‐mediated phase separation mechanisms, especially the search or screening of crucial molecules that regulate phase separation, such as kinases, will provide a theoretical evidence for validating the effectiveness of target molecules.

## CONFLICT OF INTEREST

The authors have confirmed that there are no conflicts of interest.

## Data Availability

No data are available for this article.
